# Crosstalk with Inflammatory Macrophages Shapes the Regulatory Properties of Multipotent Adult Progenitor Cells

**DOI:** 10.1155/2017/2353240

**Published:** 2017-07-12

**Authors:** Stylianos Ravanidis, Jeroen F. J. Bogie, Raf Donders, Robert Deans, Jerome J. A. Hendriks, Piet Stinissen, Jef Pinxteren, Robert W. Mays, Niels Hellings

**Affiliations:** ^1^Biomedical Research Institute/Transnational University Limburg, School of Life Sciences, Hasselt University, 3590 Diepenbeek, Belgium; ^2^Department of Regenerative Medicine, Athersys Inc., Cleveland, OH, USA; ^3^ReGenesys BVBA, Leuven, Belgium

## Abstract

Macrophages and microglia are key effector cells in immune-mediated neuroinflammatory disorders. Driving myeloid cells towards an anti-inflammatory, tissue repair-promoting phenotype is considered a promising strategy to halt neuroinflammation and promote central nervous system (CNS) repair. In this study, we defined the impact of multipotent adult progenitor cells (MAPC), a stem cell population sharing common mesodermal origin with mesenchymal stem cells (MSCs), on the phenotype of macrophages and the reciprocal interactions between these two cell types. We show that MAPC suppress the secretion of tumor necrosis factor alpha (TNF-*α*) by inflammatory macrophages partially through a cyclooxygenase 2- (COX-2-) dependent mechanism. In turn, we demonstrate that inflammatory macrophages trigger the immunomodulatory properties of MAPC, including an increased expression of immunomodulatory mediators (e.g., inducible nitric oxide synthase (iNOS) and COX-2), chemokines, and chemokine receptors. Macrophage-primed MAPC secrete soluble factors that suppress TNF-*α* release by macrophages. Moreover, the MAPC secretome suppresses the antigen-specific proliferation of autoreactive T cells and the T cell stimulatory capacity of macrophages. Finally, MAPC increase their motility towards secreted factors of activated macrophages. Collectively, these in vitro findings reveal intimate reciprocal interactions between MAPC and inflammatory macrophages, which are of importance in the design of MAPC-based therapeutic strategies for neuroinflammatory disorders in which myeloid cells play a crucial role.

## 1. Introduction

Increasing evidence indicates that stem cell transplantation harbors potential to treat neuroinflammatory disorders [1]. For instance, neural precursor cells (NPCs) and mesenchymal stem cells (MSCs) possess functional immunomodulatory and neuroprotective properties as demonstrated by attenuation of disease severity after transplantation in experimental models of central nervous system- (CNS-) associated diseases, namely, multiple sclerosis (MS) and traumatic brain injury (TBI) among others [2–5].

Macrophages and microglia are key effector cells in the pathogenesis of neuroinflammatory disorders [6, 7]. Myeloid cells are regarded to be mainly detrimental in autoimmune diseases of the CNS as they promote neuroinflammation, demyelination, and neurodegeneration [8]. Inflammatory and toxic secretions, such as tumor necrosis factor alpha (TNF-*α*), interleukin 6 (IL-6), interleukin 1 beta (IL-1*β*), and nitric oxide (NO), partially underlie the disease-promoting impact of myeloid cells in MS [8, 9]. Furthermore, myeloid cells can promote neuroinflammation through the activation of encephalitogenic T cells and by promoting their recruitment to the CNS [9, 10]. Therefore, driving myeloid cells towards an anti-inflammatory and regenerative phenotype is considered a promising strategy to halt disease progression in neuroinflammatory disorders.

Several types of adult stem cells have been shown to skew myeloid cells towards a neuroprotective phenotype. For instance, MSCs can suppress the release of inflammatory mediators such as TNF-*α* and IL-6 by macrophages, while simultaneously inducing an “M2-like” anti-inflammatory and reparative phenotype in vitro and in vivo [11–14]. Moreover, macrophages seem to shape MSCs enhancing their immunomodulatory functions and altering their migratory properties [15, 16]. These studies point towards intimate reciprocal interactions between stem cells and macrophages.

Multipotent adult progenitor cells (MAPC) are bone marrow-derived stem cells that share a common mesodermal origin with MSCs. However, compared to MSCs, MAPC show a faster expansion rate and long-term population doublings of MAPC occur without signs of replicative senescence providing sufficient quantities for future therapeutic use [17, 18]. Moreover, MAPC show superior in vitro and in vivo immune suppressive features compared to MSCs [19, 20]. Particularly, using a xenogeneic transplantation approach, human MAPC (hMAPC) induced an “M2-like” polarization of microglia and macrophages in experimental models of TBI and spinal cord injury (SCI) [21–23]. In another study, rat MAPC (rMAPC) reduced the expression of metalloproteinase 9 (MMP-9) in macrophages, thereby preventing the macrophage-mediated induction of axonal dieback in SCI [24]. Moreover, it seems that the plasticity of rMAPC is shaped when they encounter an inflammatory environment [25]. These features make MAPC an attractive alternative for stem cell transplantation in CNS disorders [21–24, 26]. However, to date, the reciprocal interactions between MAPC and myeloid cells remain to be fully characterized.

In this study, we sought to determine the in vitro reciprocal interactions between macrophages and MAPC. We show that MAPC suppress the inflammatory phenotype that macrophages acquire following lipopolysaccharide (LPS) stimulation. In parallel, macrophage-exposed MAPC acquire an enhanced T cell modulatory phenotype. Moreover, MAPC increase their motility towards the inflammatory environment of classically activated macrophages. Collectively, these in vitro findings indicate that intimate interactions between MAPC and macrophages occur, resulting in enhanced therapeutic potency of MAPC. This study warrants in vivo validation and can, in the long run, assist in appropriate tissue targeting in preclinical autologous experimental studies.

## 2. Materials and Methods

### 2.1. rMAPC Culture and Chemicals

Lewis rat-derived MAPC (rMAPC) were provided by ReGenesys BVBA (Leuven, Belgium) and maintained according to the standard protocols developed by the supplier (37°C/5% CO_2_/5% O_2_). Cells were isolated and expanded as described previously [25, 27]. rMAPC medium consisted of 60% Dulbecco's Modified Eagle medium (DMEM; Gibco, Life Technologies Europe B.V., Gent, Belgium) low glucose (1 g/L), 40% MCDB-201 medium (pH 7.2), 1X linoleic acid-bovine serum albumin, 10^−4^ M l-ascorbic acid, 0.05 *μ*M dexamethasone, 55 *μ*M 2-mercapto-ethanol (all from Sigma-Aldrich, Diegem, Belgium), 100 IU/mL penicillin and 100 *μ*g/mL streptomycin (Invitrogen, Life Technologies Europe B.V.), 1X insulin-transferrin-selenium (Lonza, Verviers, Belgium), 10 ng/mL mouse epidermal growth factor, 10 ng/mL recombinant human platelet-derived growth factor (R&D Systems, Abingdon, United Kingdom), and 10^3^ units/mL mouse leukemia inhibitory factor (Millipore, Billerica, MA, USA). Finally, medium was supplemented with 2% fetal bovine serum (FBS; Hyclone, EU approved, Cat CH30160.03). Cells were cultured in human-derived fibronectin (10 ng/mL; Sigma-Aldrich) T175 flasks (Cellstar, Greiner Bio-One, Vilvoorde, Belgium) or Petri dishes (Nunc, VWR, Leuven, Belgium) according to the purposes needed.

Prostaglandin E2 (PGE2_,_ Enzo Life Sciences, Farmingdale, NY, USA) was added on macrophages in specific experiments. PGE2 was diluted in dimethyl sulfoxide (DMSO; Sigma-Aldrich) which was used also as negative control.

### 2.2. Peritoneal Macrophages, NR8383 Cells, and Generation of Myelin Basic Protein-Specific T Cells

Macrophages were isolated from the peritoneal cavity of female Lewis rats via peritoneal lavage with 5 mM ethylenediaminetetraacetic acid (EDTA; VWR, Leuven, Belgium) solution in phosphate-buffered saline (PBS) as previously described [28]. Cells were collected and centrifuged at 1400 rpm for 10 minutes. After seeding, cells were allowed to adhere for 3 hours and then were washed twice with Roswell Park Memorial Institute- (RPMI-) 1640 (Invitrogen, Life Technologies Europe B.V.) to remove nonadherent peritoneal exudate cells. Peritoneal macrophages, as well as the alveolar macrophage-like cell line NR8383 [29], were cultured in RPMI-1640 supplemented with 10% fetal calf serum (FCS, Gibco, Life Technologies Europe B.V., Gent, Belgium) and 0.5% penicillin-streptomycin mixture (Gibco).

For the generation of myelin basic protein- (MBP-) specific T cells, the experimental model of MS, experimental autoimmune encephalomyelitis (EAE) [9], was induced in female Lewis rats (Janvier, France). Rats were injected subcutaneously with a 0.1 mL solution of 250 *μ*g/mL guinea pig MBP, 2.5 mg/mL H37RA heat-killed mycobacterium tuberculosis (Difco, Detroit, USA), and 60 *μ*L complete Freund's adjuvant (Sigma-Aldrich) in both hind paws. Ten days postimmunization, rats were sacrificed and popliteal and inguinal lymph nodes were excised. Tissues were grinded through a 70 *μ*m cell strainer to generate a single -cell suspension. MBP-specific T cells were generated as described before [30].

All animal experiments were approved by the Ethical Committee for Animal Experiments of Hasselt University.

### 2.3. Cocultures

The effect of rMAPC on the phenotype of macrophages was determined in direct and transwell cocultures. Macrophages were seeded in 24-well plates (5 × 10^5^/well), and rMAPC were added in ratios ranging from 1 : 0.5 up to 1 : 4. Cells were allowed for a preconditioning period for 24 hours, and then 100 ng/mL LPS (Sigma-Aldrich) was added for 20 hours. To evaluate the role of cyclooxygenase (COX) in the observed effects, 10 *μ*M indomethacin (Sigma-Aldrich) was added along with LPS stimulation.

### 2.4. Exposure of rMAPC to Macrophage Supernatant and Generation of Conditioned Media

Macrophages were stimulated with 100 ng/mL LPS for 12 hours, and the supernatant (SN) was collected, filtered through a 0.45 *μ*m filter, and applied to rMAPC for 12 hours. Supernatant of untreated macrophages was used as a control. To define differences in messenger ribonucleic acid (mRNA) expression, rMAPC were incubated with SN of ±LPS-activated macrophages or LPS (7.5 × 10^5^ cells/well). Exposure to the SN of LPS-activated macrophages was prolonged to 18 hours for measuring NO using the Griess reagent assay (Promega, Leuven, Belgium).

To prepare double-conditioned media (DCM), rMAPC (5 × 10^4^ cells/well), previously exposed to the SN of LPS-activated macrophages for 12 hours, were allowed to secrete soluble factors for 24 hours in 50 *μ*L macrophage medium (96-well plate). In specific experiments, rMAPC were stimulated with a mixture of recombinant rat TNF-*α*, IL-1*β*, and IL-6 (100 ng/mL each; all from Peprotech, London, UK) to partially mimic the SN of LPS-activated macrophages. This conditioned medium is designated as “licensed-” conditioned medium (LCM). Nonstimulated rMAPC provided the single-conditioned media (CM). The representation of the generation of conditioned media from rMAPC is illustrated in Supplemental Figure 1 available online at https://doi.org/10.1155/2017/2353240. All the conditioned media were collected and filtered through a 0.45 *μ*m filter.

### 2.5. Application of Soluble Factors Derived by rMAPC to Macrophages and T Cells

Macrophages (1.5 × 10^5^/well/96-well plate) were exposed to LPS (100 ng/mL) and conditioned media from rMAPC for 24 hours. After this period, soluble mediators were measured in the supernatant. In parallel, to determine mRNA expression of M1 and M2 markers, NR8383 cells (7.5 × 10^5^ cells/well/24-well plate) were seeded in the conditioned media with or without polarization stimuli. Control conditions included cells in fresh medium with M1 stimuli (100 ng/mL LPS), M2 stimuli {100 ng/mL interleukin 4 (IL-4), 150 ng/mL interleukin 13 (IL-13), and 250 ng/mL interleukin 10 (IL-10)}, or without any stimuli (M0).

To define the effect of rMAPC-derived conditioned media on antigen-specific T cells, MBP-reactive T cells (2.5 × 10^5^) and irradiated thymocytes (2.5 × 10^5^ cells/well, 3000 rad) were seeded in 96-well U-plates in CM, DCM, or LCM and were exposed to 10 *μ*g/mL MBP. Conditioned media were diluted 1 : 1 with fresh T cell medium. The consistency of T cell medium was as previously described [25]. Control conditions included T cells and thymocytes seeded in a 1 : 1 mixture of nonconditioned and T cell medium with or without MBP. After 48 hours, 1 *μ*Ci ^3^H thymidine (PerkinElmer, Waltham, MA, USA) per well was added for 18 hours. Following this period, cells were harvested (automatic cell harvester) and thymidine incorporation was measured in a *β*-plate liquid scintillation counter (PerkinElmer). Stimulation index of proliferated cells was calculated based on the individual conditions where no exogenous stimulus (MBP) was added.

### 2.6. Migration Assay

Migration of rMAPC towards the soluble factors secreted by LPS-activated macrophages was assessed using an 8 *μ*M diameter pore transwell assay. To avoid false positive migration towards increased serum concentrations [31], macrophages were seeded in macrophage medium containing 1% FCS, instead of 10%, and were stimulated with LPS for 24 hours. The conditioned medium from macrophages was seeded in the lower part of the 24-well inserts. rMAPC (3 × 10^4^ cells/well) were suspended in DMEM low glucose 1 g/L (Gibco) containing 1% FCS in the upper chamber. Nonconditioned macrophage medium supplemented with 1% FCS served as negative while rMAPC medium served as positive control [25]. Cells were allowed to migrate for 14 hours at 37°C. Then, the media in the lower chamber were removed and inserts were fixed with 4% solution of paraformaldehyde (PFA) for 20 minutes and then they were washed with PBS (Lonza) twice. Subsequently, cells were stained with 0.1% Crystal Violet (Sigma-Aldrich) solution in ethanol for 10 minutes. Cells on the top side of the insert were removed using a cotton swab. Then, inserts were washed with PBS, allowed to air dry and three pictures per well were taken. Using ImageJ software, pictures were transformed to 8-bit images and the migrated fraction is expressed as percentage of the total covered area of the well [32].

### 2.7. Antigen Recall Assay

To define the impact of conditioned media from rMAPC on the capacity of macrophages to present antigens, macrophages (5 × 10^4^/well/96-well plate) were pulsed with 25 *μ*g/mL MBP in the presence of conditioned media from rMAPC. Following 18 hours, the conditioned media were removed and 1.5 × 10^5^ carboxyfluorescein diacetate succinimidyl ester- (CFSE-; Invitrogen) labeled MBP-specific T cells (2 *μ*M) were added (T cells/macrophage ratio 3 : 1). After 4 days, T cells were collected and processed for flow cytometry. T cells were stained with rat anti-CD3 (eBioscience, Vienna, Austria) and 7 aminoactinomycin D (7AAD; R&D Systems, Abingdon, UK) to assess cell death.

### 2.8. Flow Cytometry

The effect of conditioned media from rMAPC on endocytic properties of macrophages was assessed using fluorescein isothiocyanate- (FITC-) labeled dextran beads (Sigma-Aldrich). For this purpose, following an overnight incubation with conditioned media from rMAPC, macrophages were exposed to 100 *μ*g/mL FITC-labeled dextran beads for 2 hours. Macrophages exposed to beads on ice were taken along to correct for background levels generated by spontaneous sticking of the beads to cell membranes. In separate experiments, cells were stained with anti-rat CD86 (eBioscience) and the mean FL2 signal within the CD86 gate was used to evaluate the CD86 expression. In coculture experiments, cells were stained with anti-CD11 b/c (BioLegend, San Diego, CA, USA) along with anti-CD86, and the mean expression of FL2 channel was used to measure CD86 expression.

### 2.9. Analysis of Gene Expression

RNA was isolated with RNeasy mini kit (Qiagen, Venlo, The Netherlands) and was transcribed to complementary deoxyribonucleic acid (cDNA) using the Quanta kit (VWR, Leuven, Belgium) following the manufacturer's instructions. Semiquantitative real-time polymerase chain reaction (qPCR) was performed to detect changes in gene expression. Reactions were performed in a StepOnePlus™ Real-Time PCR System (Applied Biosystems) in micro AMP Fast Optical 96-well reaction plates in a total volume of 10 *μ*L per reaction. Master mix consisted of 1x Fast SYBR green master mix (Applied Biosystems), 10 mM of each primer (designed with Primer 3 [33]; Eurogentec, Liege, Belgium), nuclease-free water, and 12.5 ng of cDNA template. Following the amplification, melting curve analysis was performed to test the specificity of the qPCR products. The primer sequences used are listed in Supplemental Table 1. The 2^−ΔΔCt^ method was used to relatively quantify the expression of each gene [34] while data were normalized to the most stable reference genes for each experiment following geNorm analysis [35].

### 2.10. Measurement of Soluble Mediators and Nitrite Formation

Cytokines were measured with sandwich enzyme-linked immunosorbent assay (ELISA). TNF-*α* (eBioscience) and IL-6 (R&D Systems, Minneapolis, MN, USA) ELISA were used following the manufacturer's instructions, and absorbance was measured at 450 nm using a spectrophotometer (Bio-Rad Benchmark, Bio-Rad Laboratories, Hercules, CA, USA). The presence of nitrite was measured using Griess reagent system (Promega, Leuven, Belgium) following the manufacturer's instructions, and absorbance was measured at 540 nm.

### 2.11. Statistical Analysis

Data were analyzed with the GraphPad Prism version 5.00 for Windows (GraphPad Software, San Diego, California, USA, www.graphpad.com) and are presented as mean ± SEM. D'Agostino and Pearson omnibus normality test was used to evaluate whether the data are following normal distribution. For comparisons between two groups, unpaired Student's *t*-test or Mann–Whitney *U* test was used. One-way analysis of variance (ANOVA) followed by Dunnett's multiple comparison test or Kruskal-Wallis followed by Dunn's multiple comparison test was used, depending on whether the data were parametric or nonparametric, for comparisons between multiple groups. Differences with *p* value ≤0.05 were considered significant.

## 3. Results

### 3.1. rMAPC Release NO When Cocultured with Inflammatory Macrophages

**Figure 1:**
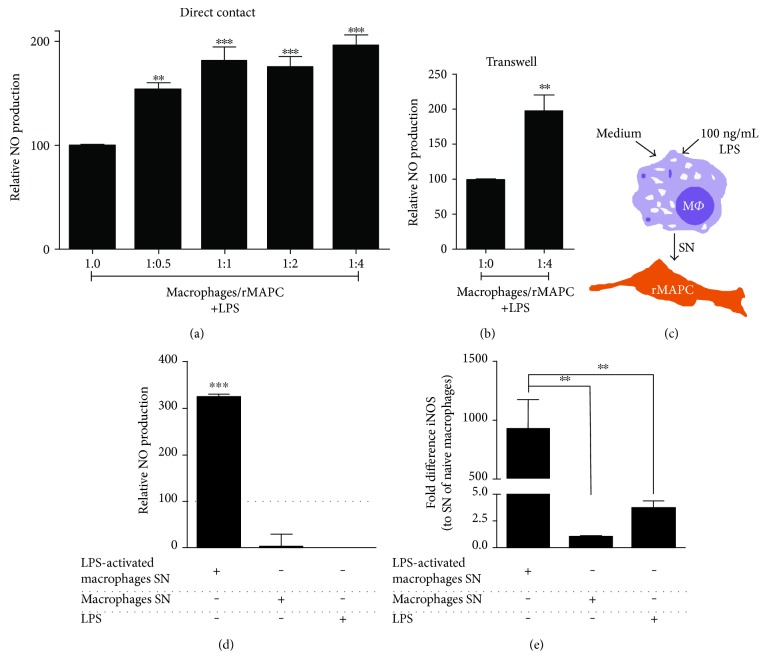
rMAPC increase NO production in the presence of inflammatory macrophages. (a) Direct contact cocultures (*N* = 7 experiments) of macrophages with rMAPC in increasing ratios (1 : 0 to 1 : 4) and (b) transwell cocultures of macrophages with rMAPC (1 : 4, *N* = 6  experiments), supplemented with LPS. The results are shown as percentage of nitrite levels normalized to the positive control (1 : 0 +LPS) with duplicates per experiment. Asterisks (^∗^) indicate statistical significant difference with positive control (1 : 0 +LPS). (c) Schematic illustration of generation of LPS-activated SN (M*Φ*; macrophages). (d) rMAPC treated with SN of LPS-activated macrophages, SN of naïve macrophages or LPS alone (*N* = 4 experiments). The results are shown as percentage of nitrite levels normalized to the positive control (pure s/n from activated macrophages, dotted line). Asterisks (^∗^) indicate statistical significant difference with positive control. (e) iNOS mRNA expression of rMAPC treated with SN of LPS-activated macrophages, SN of naïve macrophages or LPS (*N* = 5  experiments). The results are shown as fold differences in comparison to SN of naïve macrophages. Asterisks (^∗^) indicate statistical significant differences. Mean values ± SEM. Data were analyzed with one-way ANOVA followed by Dunnett's multiple comparison test or with Kruskal-Wallis test followed by Dunn's multiple comparison test for nonparametric dat (^∗∗^*p* ≤ 0.01 and ^∗∗∗^*p* ≤ 0.001).

ΝΟ has multimodal functions in neuroinflammatory disorders. While high levels of NO can be detrimental for oligodendrocytes and neurons [36], NO can also inhibit the proliferation of autoreactive T cells [30]. In this part of the study, we sought to determine NO release in cocultures with rMAPC and macrophages. Our data show that NO levels were increased in both direct and transwell cocultures of inflammatory macrophages and rMAPC (Figures 1(a) and 1(b)). Nonstimulated macrophages did not release detectable levels of NO (data not shown). To determine the cellular source of NO in cocultures, rMAPC were incubated with SN of LPS-activated macrophages (Figure 1(c)). We observed that rMAPC released NO above the levels already present in the supernatant due to LPS stimulation of macrophages (Figure 1(d)). In line with this finding, rMAPC showed increased mRNA expression of iNOS upon exposure to SN of LPS-activated macrophages compared to rMAPC stimulated with SN of nonstimulated macrophages or LPS alone (Figure 1(e)).

### 3.2. rMAPC Suppress the Release of TNF-*α* by Macrophages Partially through a COX-2-Dependent Mechanism

**Figure 2:**
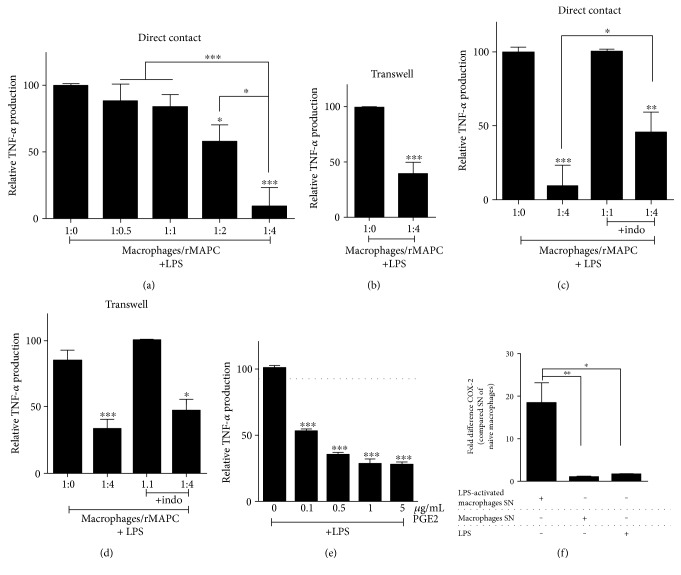
rMAPC suppress TNF-*α* release by macrophages in a COX-2-dependent mechanism. (a) Direct contact cocultures (*N* = 9 experiments) of macrophages with rMAPC in increasing ratios (1 : 0 to 1 : 4) and (b) transwell cocultures of macrophages with rMAPC (1 : 4, *N* = 4 experiments), supplemented with LPS. The results are shown as percentage of TNF-*α* levels normalized to the positive control (1 : 0 +LPS) with duplicates per experiment. Asterisks (^∗^) indicate statistical significant difference with positive control. (c) Direct contact (*N* = 7 experiments) and (d) transwell coculture (*N* = 4 experiments) of macrophages with rMAPC supplemented with LPS (1 : 4) and indomethacin. The results are shown as percentage of TNF-*α* levels normalized to the positive control (1 : 0 +LPS + indo) with duplicates per experiment. Asterisks (^∗^) indicate statistical significant difference with positive control. (e) Effect of PGE2 (0 to 5 *μ*g/mL) on TNF-*α* release by macrophages after LPS stimulation (*N* = 4 experiments with triplicates per experiment). The results are shown as percentage of TNF-*α* levels normalized to the positive control (0 *μ*g/mL +LPS). Asterisks (^∗^) indicate statistical significant difference with positive control. Mean values of dimethyl sulfoxide are shown as solvent of PGE2 (dotted line). (f) Cyclooxygenase-2 (COX-2) mRNA expression in rMAPC treated with SN of LPS-activated macrophages, SN of naïve macrophages or LPS (*N* = 5 experiments). The results are shown as fold differences in comparison to SN of naïve macrophages. Asterisks (^∗^) indicate statistical significant differences. Mean values ± SEM. Data were analyzed with one-way ANOVA followed by Dunnett's multiple comparison test. ^∗^*p* ≤ 0.05, ^∗∗^*p* ≤ 0.01, and ^∗∗∗^*p* ≤ 0.001.

Adult stem cells hold potential in suppressing the inflammatory properties of classically activated macrophages or even direct them towards an “M2-like” phenotype [12, 14, 37]. Direct and transwell cocultures of rMAPC and LPS-stimulated macrophages were established to determine the effect of rMAPC on the release of inflammatory mediators by macrophages. rMAPC suppressed the release of TNF-*α* by activated macrophages in a dose-dependent manner (Figure 2(a)). Nonstimulated macrophages did not produce detectable levels of TNF-*α* (data not shown). A similar inhibition of TNF-*α* release was observed when cell contact between rMAPC and macrophages was blocked by using transwell inserts (Figure 2(b)). rMAPC did not produce detectable levels of TNF-*α* following treatment with SN of ±LPS-activated macrophages (data not shown).

Prostaglandin E2 (PGE2) is well known to modulate the phenotype of macrophages. Interestingly, the expression of its rate-limiting enzyme involved in the generation of PGE2, cyclooxygenase 2 (COX-2), is induced in MSCs and MAPC under inflammatory conditions [12, 14, 25]. By using an inhibitor of COX-2, we show that the reduction of TNF-*α* release by macrophages in direct contact cocultures with rMAPC is partially abrogated (Figure 2(c)). In contrast, inhibition of COX-2 did not significantly counteract the reduced levels of TNF-*α* in transwell cocultures (Figure 2(d)). To confirm the capacity of PGE2 to reduce TNF-*α* release, macrophages were exposed to PGE2 along with LPS stimulation. We demonstrate that PGE2 suppressed the release of TNF-*α* by LPS-stimulated macrophages in a dose-dependent manner (Figure 2(e)). Finally, we found that rMAPC have an increased mRNA expression of COX-2, but not COX-1, when exposed to the SN of LPS-activated macrophages compared to rMAPC treated with SN from naïve macrophages or LPS alone (Figure 2(f) and data not shown). The latter finding strongly suggests that macrophage-driven expression of COX-2 by rMAPC is involved in the regulation of the macrophage phenotype.

### 3.3. rMAPC Increase IL-6 Secretion in Response to Activated Macrophages

**Figure 3:**
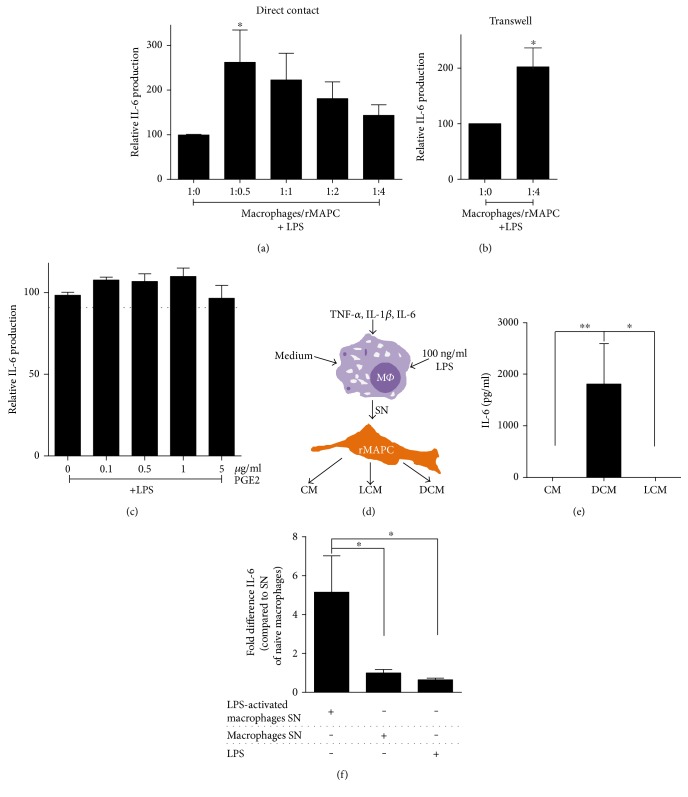
Macrophage-primed rMAPC increase their IL-6 expression. (a) Direct contact cocultures (*N* = 6 experiments) of macrophages with rMAPC in increasing ratios (1 : 0.5 to 1 : 4) and (b) transwell cocultures of macrophages with rMAPC (1 : 4, *N* = 4 experiments), supplemented with LPS. The results are shown as percentage of IL-6 levels normalized to the positive control (1 : 0 +LPS) with duplicates per experiment. Asterisks (^∗^) indicate statistical significant difference with positive control. (c) Effect of PGE2 (0 to 5 *μ*g/mL) on TNF-*α* release by macrophages after LPS stimulation (*N* = 4 experiments with triplicates per experiment). The results are shown as percentage of IL-6 levels normalized to the positive control (0 *μ*g/mL +LPS). Asterisks (^∗^) indicate statistical significant difference with positive control. Mean values of dimethyl sulfoxide are shown as solvent of PGE2 (dotted line). (d) Schematic illustration of generation of rMAPC-conditioned media (SN; supernatant, M*Φ*; macrophages). (e) IL-6 measurement in rMAPC-derived conditioned media (*N* = 6 experiments) following different stimuli. IL-6 levels are shown as pg/mL. Asterisks (^∗^) indicate statistical significant difference between the different conditioned media. (f) IL-6 mRNA expression in rMAPC treated with SN of LPS-activated macrophages, SN of naïve macrophages or LPS (*N* = 5 experiments). The results are shown as fold differences in comparison to SN of naïve macrophages. Asterisks (^∗^) indicate statistical significant differences. Mean values ± SEM. Data were analyzed with one-way ANOVA followed by Dunnett's multiple comparison test (^∗^*p* ≤ 0.05, ^∗∗^*p* ≤ 0.01).

In contrast to TNF-*α* and similar to NO, levels of IL-6 were increased in both direct and transwell cocultures of macrophages and rMAPC (Figures 3(a) and 3(b)). Application of COX-2 inhibitor did not have any impact on IL-6 secretion (data not shown). In line with this finding, PGE2 did not affect the release of IL-6 by macrophages as seen with TNF-*α* (Figure 3(c)). To define the cellular source of IL-6 in cocultures, rMAPC were incubated with SN of LPS-activated macrophages for a short period. Then, new medium was applied for 24 hours and this was used for measuring IL-6 levels (Figure 3(d)). Macrophage-primed rMAPC increased their IL-6 secretion (DCM), while naïve or cytokine-primed rMAPC did not produce detectable levels of IL-6 (Figure 3(e)). Also, the mRNA expression of IL-6 in rMAPC was increased compared to rMAPC treated with SN of macrophages or LPS alone (Figure 3(f)). These findings point towards rMAPC as the cellular source of the additional IL-6 release in LPS-stimulated cocultures. Of interest, no IL-6 production was observed in nonstimulated cocultures, indicating that LPS stimulation is necessary for macrophages to induce IL-6 release by rMAPC.

### 3.4. Macrophage-Primed rMAPC Secrete Factors That Modulate the Macrophage Phenotype

**Figure 4:**
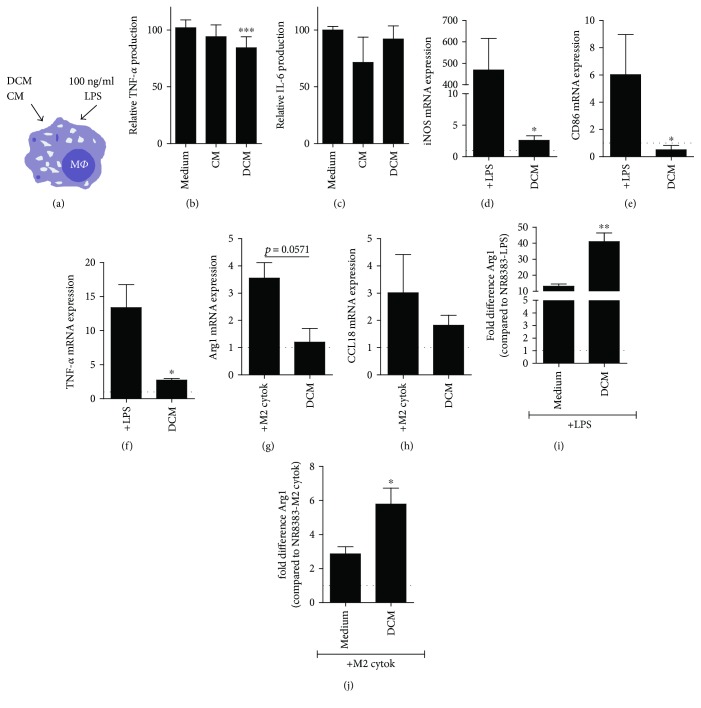
Soluble factors derived by macrophages-primed rMAPC modulate macrophages phenotype. (a) Schematic illustration of application of rMAPC-derived conditioned media to macrophages (M*Φ*). (b) TNF-*α* (*N* = 6 independent experiments with triplicates per experiment) and (c) IL-6 (*N* = 3 experiments with triplicates per experiment) secretion levels of macrophages treated with LPS in the presence of rMAPC-derived conditioned media. The results are presented as percentage of to the positive control (medium +LPS). Asterisks (^∗^) indicate statistical significant difference with positive control. (d–h) NR8383 mRNA expression of M1 and M2 polarization markers seeded in DCM (*N* = 5 experiments). iNOS (d), CD86 (e), and TNF-*α* (f) are compared to their respective positive control (NR8383 +LPS) while Arg1 (g) and CCL18 (h) to NR8383 +M2 cytokines (IL-4/IL-10/IL-13). The results are presented as fold differences to nonstimulated NR8383 cells (dotted line). (i-j) NR8383 mRNA expression of Arg1 seeded in DCM supplemented with polarization stimulus, +LPS (i) and +M2 cytokines (j). The results are presented as fold differences to nonstimulated NR8383 cells (dotted line). Asterisks (^∗^) indicate statistical significant difference with positive control in each case (NR8383 +LPS or NR8383 +M2 cytokines). Relative expressions were normalized against the expression of hydroxymethylbilane synthase (HMBS) and b-actin. Mean values ± SEM. Data were analyzed with one-way ANOVA followed by Dunnett's multiple comparison test or with Kruskal-Wallis test followed by Dunn's multiple comparison test for nonparametric data. Two group comparisons were made with unpaired Student's *t*-test (^∗^*p* ≤ 0.05, ^∗∗^*p* ≤ 0.01, and ^∗∗∗^*p* ≤ 0.001).

Increasing evidence indicates that inflammatory conditions promote the immunomodulatory features of stem cells [38]. To explore whether rMAPC primed by inflammatory macrophages reciprocally impact macrophage physiology, double-conditioned medium was generated. For this purpose, rMAPC were initially triggered with SN of LPS-activated macrophages, simulating the inflammatory mediators released by “M1-like” macrophages in immune-mediated diseases, and then allowed to secrete factors in their supernatant (Supplemental Figure 1). Macrophages exposed to the double-conditioned media from rMAPC (DCM) (Figure 4(a)) secreted less TNF-*α* following LPS stimulation (Figure 4(b)). This effect was not due to increased cell death of macrophages (not shown). Single-conditioned media (CM) showed no effect, indicating the necessity of priming of rMAPC to regulate the TNF-*α* secretion levels by macrophages. IL-6 levels were not affected in any condition (Figure 4(c)). To detect the effects of rMAPC-derived conditioned media on mRNA expression of polarization markers, we used a macrophage-like cell line, NR8383. DCM did not induce spontaneous expression of M1 (iNOS, CD86, and TNF-*α*; Figures 4(d), 4(e), and 4(f)) or M2 markers {arginase 1 (Arg1) and C-C motif ligand 18 (CCL18); Figures 4(g) and 4(h)} in NR8383 cells. However, when NR8383 cells were simultaneously incubated in DCM and were stimulated with either LPS or M2-inducing cytokines (IL-4/IL-10/IL-13), we observed an increased mRNA expression of Arg1, the prototypical marker for M2 macrophages (Figures 4(i) and 4(j)). An increased mRNA expression of CCL18 by NR8383 cells incubated in DCM was observed only following LPS stimulation (data not shown).

Collectively, these results indicate that the regulatory activities of rMAPC towards macrophages are shaped by the factors released by macrophages.

### 3.5. Macrophage-Primed rMAPC Suppress Autoreactive T Cell Proliferation

**Figure 5:**
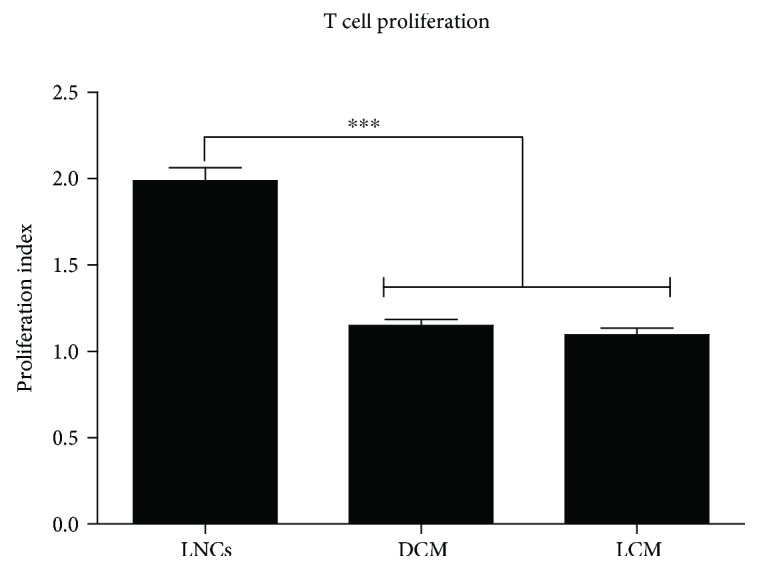
rMAPC suppress antigen-specific T cell proliferation. Myelin basic protein-(MBP-) specific T cells were seeded in rMAPC-derived conditioned media and were stimulated with MBP. Proliferation index was calculated based to conditions without MBP (not shown). Mean values ± SEM are from 3 independent experiments, with triplicates per experiment. Data were analyzed with one-way ANOVA followed by Dunnett's multiple comparison test ^∗∗∗^*p* ≤ 0.001.

The proliferation of autoreactive T cells is closely associated with disease severity in neuroinflammatory conditions [39, 40]. As proinflammatory priming is reported to enhance the capacity of MSCs to suppress T cell proliferation [25, 38], we assessed whether macrophage-mediated priming of rMAPC also enhances their capacity to suppress myelin-reactive T cells. For this purpose, MBP-reactive T cells were exposed to cognate antigen and double-conditioned media of rMAPC. Additionally, MBP-reactive T cells were exposed to conditioned medium of rMAPC that were primed with TNF-*α*, IL-1*β*, and IL-6, cytokines that are typically secreted by inflammatory macrophages [41]. Our data indicate that cytokine- (LCM) and macrophage- (DCM) primed rMAPC suppress the proliferation of MBP-reactive T cells to a similar extent (Figure 5).

### 3.6. rMAPC-Derived Soluble Factors Interfere with the Antigen Presentation Capacity of Macrophages

**Figure 6:**
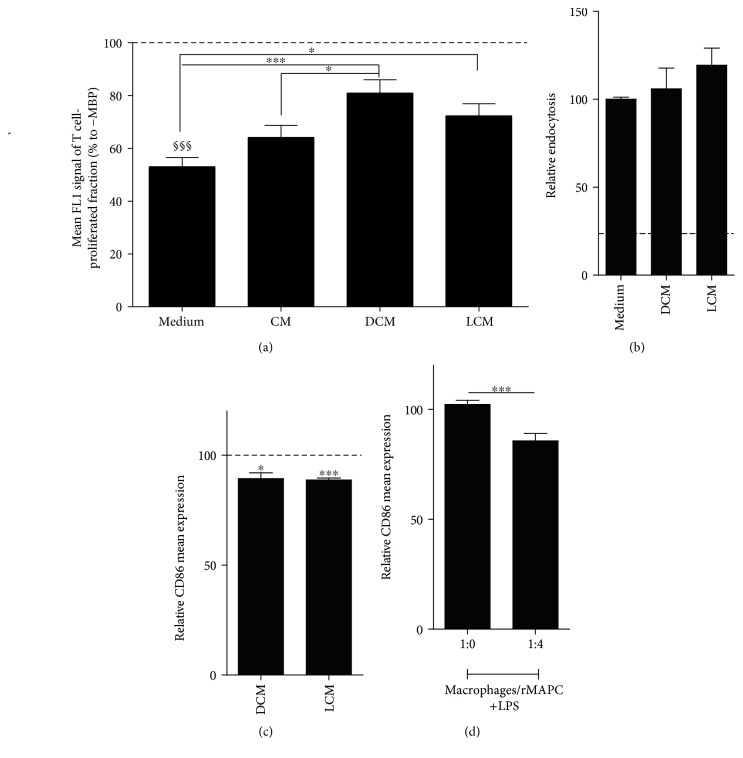
rMAPC suppress antigen presenting features of macrophages. (a) Coculture of macrophages and T cells following macrophages' pulsing with MBP in specified conditioned media from rMAPC or in their own medium (*N* = 3 experiments with quadruplicates per experiment). T cell proliferation was estimated based to the mean FL1 signal of the proliferated fraction of carboxyfluorescein diacetate succinimidyl ester (CFSE) labeling. The results are shown as percentages of negative control (macrophages cocultured with T cells without prior pulsing of MBP, dotted line). Paragraph signs (§) indicate statistical significant difference between positive control (pulsed macrophages with MBP in fresh medium cocultured with T cells) and negative control. Asterisks (^∗^) indicate statistical significant difference with positive control. (b) Endocytosis of FITC-labelled beads of macrophages when incubated with designated media. The results are shown as percentages of positive control (endocytosis in fresh medium). Background levels of endocytosis are shown in *dotted line*. (c) Mean of CD86 expression of macrophages incubated with designated media (*N* = 4 experiments with duplicates per experiment). The results are presented as percentages of positive control (macrophages in fresh medium) (dotted line). Asterisks (^∗^) indicate statistical significant differences with positive control. (d) Mean of CD86 expression of CD11b/c/CD86 fraction of coculture of macrophages and rMAPC (1 : 4) treated with LPS (*N* = 4 experiments with quadruplicates per experiment). The results are presented as percentages of positive control (1 : 0 +LPS). Asterisks (^∗^) indicate statistical significant difference with positive control. Mean values ± SEM. Data were analyzed with one-way ANOVA followed by Dunnett's multiple comparison test or with Kruskal-Wallis test followed by Dunn's multiple comparison test for nonparametric data. ^§§§^*p* ≤ 0.001, ^∗^*p* ≤ 0.05, and ^∗∗∗^*p* ≤ 0.001.

Macrophages are specialized antigen-presenting cells. The processing and the subsequent presentation of myelin antigens by macrophages promote neuroinflammation and disease progression [42]. To define whether soluble factors derived from primed rMAPC affect T cell stimulatory capacity of macrophages, macrophages were pulsed with MBP in the presence of conditioned media prior to coculture with MBP-reactive T cells. Macrophages that were exposed to double-conditioned media (DCM) and licensed-conditioned medium (LCM) showed a reduced capacity to stimulate T cells compared to macrophages exposed to control condition (Figure 6(a)). Double- or licensed-conditioned media did not affect the endocytic ability of macrophages (Figure 6(b)). This finding suggests that a reduced uptake of MBP does not underlie the impaired capacity of macrophages to stimulate T cells. Interestingly, macrophages cultured in DCM and LCM showed a reduced surface expression of CD86 (Figure 6(c)). Similarly, macrophages showed a decreased surface expression of CD86 in direct contact coculture with rMAPC following LPS stimulation (Figure 6(d)). These findings suggest that rMAPC affect the T cell stimulatory capacity of macrophages by reducing the expression of costimulatory molecules [6, 7].

### 3.7. rMAPC Motility Is Increased towards Inflammatory Mediators Secreted from Macrophages

**Figure 7:**
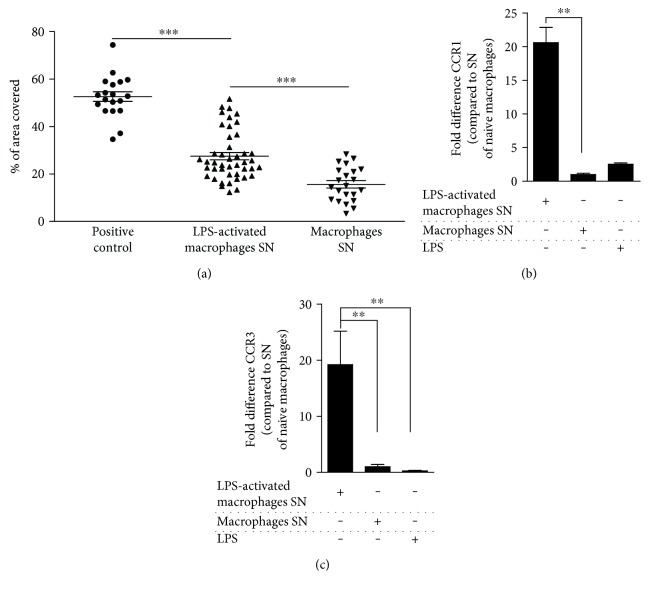
rMAPC acquire a migratory phenotype in response to SN of LPS-activated macrophages. (a) rMAPC migration towards SN of ±LPS-activated macrophages. The results are presented as percentage of area covered from the migrated fraction. A positive control rMAPC medium was used. Asterisks (^∗^) indicate statistical significant differences. (positive control versus SN of naïve macrophages; *p* < 0.001). Mean values ± SEM are from 7 independent experiments, with duplicates per experiment. (b) CCR1 and (c) CCR3 mRNA expression in rMAPC treated with SN of LPS-activated macrophages, SN of naïve macrophages, or LPS (*N* = 5 experiments). The results are shown as fold differences in comparison to the SN of naïve macrophages. Mean values ± SEM. Asterisks (^∗^) indicate statistical significant differences. Data were analyzed with one-way ANOVA followed by Dunnett's multiple comparison test. ^∗∗^*p* ≤ 0.01 and ^∗∗∗^*p* ≤ 0.001.

Migration towards inflammatory gradients released by the cells present at the site of tissue injury is an important feature of adult stem cells in the context of neuroinflammation [15, 25]. We observed an enhanced migration of rMAPC towards inflammatory macrophage-conditioned medium as compared to conditioned medium derived by nonstimulated macrophages (Figure 7(a)). An enhanced expression of C-C motif chemokine receptors, CCR1 and CCR3, on rMAPC may account for the observed increase in migration of rMAPC (Figures 7(b) and 7(c)). CCR1 and CCR3 are the receptors of CCL5, which is a chemokine that is typically secreted by classically activated macrophages [10]. These observations suggest that rMAPC increase their motility towards inflammatory gradients generated by activated macrophages. Moreover, rMAPC demonstrated increased mRNA expression of CCL2, CCL5, and CXCL10, after exposure to SN of LPS-activated macrophages (Supplemental Figure 2). Overall, these results indicate that rMAPC's exposure to secretome of classically activated macrophages may assist the establishment of interactions with the immune cells in vivo by altering their migratory and chemoattractive phenotype.

## 4. Discussion

Stem cell transplantation represents a promising therapeutic approach to treat neuroinflammatory and neurodegenerative disorders. Upon transplantation in inflammatory CNS disorders such as MS, TBI, SCI, and stroke, stem cells are likely to encounter myeloid cells in both the CNS and periphery. Myeloid cells are key effector cells in these disorders [6, 9, 43, 44]. Skewing myeloid cells towards a less inflammatory phenotype is considered to be a promising therapeutic strategy. In this study, we show that macrophages and rMAPC, a similar but functionally different adherent stem cell population than MSCs with superior features, closely interact thereby affecting each other's inflammatory and migratory phenotype. In particular, we found that rMAPC dampen the inflammatory features of classically activated macrophages. Vice versa, rMAPC acquired an immunomodulatory and migratory phenotype when exposed to soluble factors released by inflammatory macrophages. These syngeneic reciprocal interactions between rMAPC and macrophages may suppress features of neuroinflammation and therefore provide further incentive to use rMAPC to treat neuroinflammatory disorders.

Macrophages secrete a plethora of inflammatory mediators that promote neuroinflammation and neurodegeneration. TNF-*α* is highly expressed by both parenchymal and circulating myeloid cells in neuroinflammation [6] and is well known to promote neurodegeneration [45]. In line with previous studies using MSCs, our data indicate that rMAPC suppress the secretion of TNF-*α* by LPS-stimulated macrophages [11, 12]. Separation of rMAPC and macrophages did not abrogate the suppression of TNF-*α* secretion, indicating that soluble factors produced by rMAPC decrease the release of TNF-*α* by macrophages. Upon transplantation of rMAPC, this reduced expression of TNF-*α* may decrease oligodendrocytes damage within CNS and thus reduce disease progression [45, 46].

By using an inhibitor for COX-2, the rate-limiting enzyme in the formation of PGE2, we further provide evidence that rMAPC partially modulate TNF-*α* secretion by macrophages in a COX-2-dependent manner. In line with this finding, activated macrophage-conditioned medium markedly increased the mRNA levels of COX-2 in rMAPC and PGE2 administration decreased the release of TNF-*α* by macrophages. Previous studies indicate that PGE2 is crucial in mediating the immunosuppressive features of MSCs and MAPC [12, 25, 47, 48]. However, in our experiments, the levels of TNF-*α* were not completely restored following inhibition of COX-2, indicating that other mechanisms may play a synergistic role in the observed effect. These mechanisms could include IL-6, IL-10, and granulocyte macrophage colony-stimulating factor (GM-CSF) [49, 50]. Moreover, the levels of TNF-*α* were not restored when COX-2 was inhibited in transwell assays. This suggests that other synergistic mechanisms assist in the suppression of macrophage inflammatory phenotype, such as the binding of integrins to their corresponding receptors on the surface of macrophages [51]. In addition, the immunosuppressive action of PGE2 seems to be enhanced when rMAPC and macrophages are in close proximity [52]. Albeit other studies showed that PGE2 also suppresses IL-6 production by mouse peritoneal macrophages [12], PGE2 did not affect the secretion of IL-6 by macrophages in our experiments. This could be due to the increased IL-6 levels from rMAPC in our cocultures, which disguises the suppression of the IL-6 by macrophages. The impact of rMAPC on IL-6 secretion by macrophages should be further investigated. Eventually, complete abrogation of COX-2 mRNA expression, for example, with the use of siRNA, could provide a better understanding on the role of PGE2 in the MAPC-mediated immunosuppression.

Our data further indicate that soluble factors released by inflammatory macrophages promote IL-6 secretion by rMAPC in cocultures. It has been reported that macrophage-associated MSCs increase their IL-6 secretion [15]. However, while MSCs increase IL-6 secretion by macrophages [11, 50], rMAPC did not impact IL-6 production by LPS-stimulated macrophages in our experiments. IL-6 has a bimodal role as it can have both a pro- and anti-inflammatory effect. IL-6 controls the cascade of proinflammatory responses while it is also necessary for wound-healing processes in immunosuppressed mice based on its ability to induce “M2-” polarized macrophages [53–55]. Since IL-6 has been proposed as key molecule for MSCs to promote the polarization of macrophages towards an “M2” phenotype and PGE2 upregulation in MSCs is IL-6 dependent, its role has to be further explored [50, 56]. Overall, IL-6 secretion by rMAPC in inflammatory environment signifies their wide arsenal of immunomodulatory mechanisms that is triggered in events associated with immune activation.

In inflammatory disorders, macrophages and microglia secrete proinflammatory cytokines that can potentially enhance the secretion of immunomodulatory molecules by adult stem cell types [16, 56]. We found an increased secretion of NO by rMAPC, as well as elevated mRNA abundance of iNOS in rMAPC upon treatment with medium derived from inflammatory macrophages. We have previously demonstrated that rMAPC increase their immunomodulatory and neuroprotective properties when they encounter a neuroinflammatory environment [25]. Notably, apart from being immunomodulatory [25, 30, 38], NO can induce neuronal and oligodendrocyte damage [57]. Although rMAPC did not increase their expression of other proinflammatory cytokines, such as TNF-*α*, and did not skew macrophages towards an inflammatory phenotype, we cannot rule out the induction of a proinflammatory “MSC1-like” phenotype in rMAPC [58].

Aside from suppressing the inflammatory phenotype of macrophages, factors secreted by macrophage-primed and cytokines-primed rMAPC also intervened with the T cell stimulatory capacity of macrophages potentially by suppressing the expression of the costimulatory molecule CD86. Of note, cytokine-primed rMAPC-conditioned medium did not impact the T cell stimulatory capacity of macrophages to the same extent as macrophage-primed conditioned medium, while it induced greater suppression of CD86 surface expression. This indicates that other mediators than those used in recombinant form (TNF-*α*, IL-1*β*, and IL-6) contribute to the observed effects. Moreover, it suggests that additional mechanisms may mediate the effect of rMAPC on macrophage-associated T cell stimulatory activity beyond suppression of costimulatory molecules. Suppression of scavenger receptors, such as CD36 and CD14, that are involved in the recognition of antigens may be one additional mechanism and deserves further investigation as it has already been demonstrated for MSCs [59].

Furthermore, we observed that soluble factors released by macrophage-primed rMAPC directly suppressed antigen-specific T cell proliferation to the same extent as cytokine-primed rMAPC. Previous findings demonstrated the need for MAPC to interact with monocytes in order to suppress homeostatic proliferation of T cells [60]. This effect was attributed to IL-1*β*-dependent secretion of PGE2 by hMAPC. In line with this finding, we have previously found that IL-1*β* increases COX-2 mRNA in rMAPC [25]. Overall, we conclude that these immunoregulatory mechanisms can be triggered by activated macrophages while collectively these observations confirm the notion that “licensing” of MAPC enhances their paracrine effects [25, 58, 61] and renders them as an ideal cell type for transplantation in neuroinflammatory conditions.

Migratory and chemoattractive properties of transplanted cells towards myeloid cells are of paramount importance for them to mediate their immunomodulatory properties [62]. We observed that macrophages release soluble molecules that attract rMAPC and increase the expression of chemokines and chemokine receptors in rMAPC. The increase of CCR1 and CCR3 agrees with the observation that their expression is induced by TNF-*α* and IL-1*β* on rMAPC [25]. This result is useful for the direct contact interactions or the effectiveness of paracrine mechanisms [63].

## 5. Conclusions

**Figure 8:**
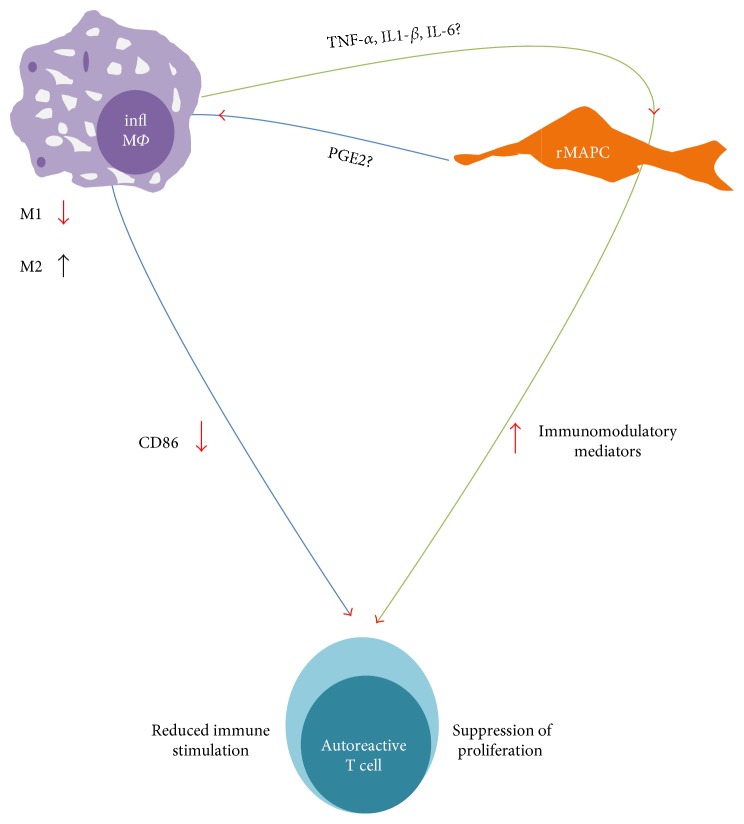
Schematic representation of reciprocal effects between rMAPC and macrophages. Inflammatory macrophages (infl M*Φ*) secrete vast amounts of proinflammatory cytokines (e.g., TNF-*α*, IL-1*β*, and IL-6) that prime rMAPC. rMAPC acquire enhanced immunoregulatory properties secreting immunomodulatory mediators that suppress autoreactive T cell proliferation (green trajectory). Moreover, immunoregulatory properties of rMAPC may suppress the M1 phenotype of M*Φ* or even induce a shift towards an M2 phenotype via the upregulation of PGE2 for instance. Effects of rMAPC secretome may lead to impaired stimulatory capacity of M*Φ* towards T cells via the downregulation of expression of surface molecules (e.g., CD86) resulting in suppressed autoreactive T cell proliferation (blue trajectory).

Macrophages have a bimodal action in autoimmune-mediated CNS diseases; while demyelinating incidents of the CNS drive macrophages and microglia activation thus contributing to the disease pathogenesis [6, 64, 65], macrophage clearance of myelin debris facilitates remyelination and reshapes their morphology towards acquisition of an anti-inflammatory and neuroprotective phenotype [30, 66–68]. Therefore, it is of high importance to elucidate how MAPC modulate the features of myeloid cell types since intravenously transplanted cells are likely to be localized in close proximity with myeloid cells in the CNS and periphery [37, 61, 69]. Overall, we showed that inflammatory macrophages are able to regulate the immunomodulatory properties of rMAPC in vitro, thereby warranting confirmatory in vivo studies. A hypothetical triangle in which inflammatory macrophages and MAPC exert reciprocal effects, leading to decreased T cell proliferation either due to paracrine effects of MAPC or due to the macrophages' reduced ability to contribute to the continuation of immunopathogenesis, should be considered in the design of experimental cell therapy schemes and is the novelty of this study (Figure 8). The efficient adaptation of MAPC in this microenvironment would favor their use in targeting myeloid cell-mediated neuroinflammation which is considered an important step on ongoing MAPC-based clinical trials [70].

## Supplementary Material

Supplemental figures Supplemental Figure 1: Schematic illustration of generation of conditioned media from rMAPC and application to macrophages (MΦ). Supplemental Figure 2: Macrophage-primed rMAPC increase mRNA expression of chemokines. *CCL2, CCL5 and CXCL10* mRNA expression in rMAPC treated with SN of LPS-activated macrophages, SN of naïve macrophages (-LPS-SN) or LPS. Results are shown as fold differences in comparison to SN of naïve macrophages. Expression of target genes was normalized against expression of *14-3-3 protein zeta/delta* (*YWHAZ*) and *TATA-binding protein* (*TBP*). Mean values ± SEM are from 5 experiments. Asterisks (∗) indicate statistical significant difference with SN of naïve macrophages. Data were analyzed with one way ANOVA followed by Dunnett's multiple comparison test. ∗p≤0.05, ∗∗p≤0.01 and ∗∗∗p≤0.001.

## References

[1] Martino G., Franklin R. J., Baron Van Evercooren A., Kerr D. A., Stem Cells in Multiple Sclerosis (STEMS) Consensus Group (2010). Stem cell transplantation in multiple sclerosis: current status and future prospects. *Nature Reviews Neurology*.

[2] Zhang J., Li Y., Chen J. (2005). Human bone marrow stromal cell treatment improves neurological functional recovery in EAE mice. *Experimental Neurology*.

[3] Ravanidis S., Poulatsidou K. N., Lagoudaki R. (2015). Subcutaneous transplantation of neural precursor cells in experimental autoimmune encephalomyelitis reduces chemotactic signals in the central nervous system. *Stem Cells Translational Medicine*.

[4] Pluchino S., Martino G. (2008). The therapeutic plasticity of neural stem/precursor cells in multiple sclerosis. *Journal of the Neurological Sciences*.

[5] Laroni A., Novi G., Kerlero de Rosbo N., Uccelli A. (2013). Towards clinical application of mesenchymal stem cells for treatment of neurological diseases of the central nervous system. *Journal of Neuroimmune Pharmacology*.

[6] Bogie J. F., Stinissen P., Hendriks J. J. (2014). Macrophage subsets and microglia in multiple sclerosis. *Acta Neuropathologica*.

[7] Kouwenhoven M., Teleshova N., Ozenci V., Press R., Link H. (2001). Monocytes in multiple sclerosis: phenotype and cytokine profile. *Journal of Neuroimmunology*.

[8] Hendriks J. J., Teunissen C. E., de Vries H. E., Dijkstra C. D. (2005). Macrophages and neurodegeneration. *Brain Research. Brain Research Reviews*.

[9] Jiang Z., Jiang J. X., Zhang G. X. (2014). Macrophages: a double-edged sword in experimental autoimmune encephalomyelitis. *Immunology Letters*.

[10] Mantovani A., Sica A., Sozzani S., Allavena P., Vecchi A., Locati M. (2004). The chemokine system in diverse forms of macrophage activation and polarization. *Trends in Immunology*.

[11] Kim J., Hematti P. (2009). Mesenchymal stem cell-educated macrophages: a novel type of alternatively activated macrophages. *Experimental Hematology*.

[12] Maggini J., Mirkin G., Bognanni I. (2010). Mouse bone marrow-derived mesenchymal stromal cells turn activated macrophages into a regulatory-like profile. *PLoS One*.

[13] Nakajima H., Uchida K., Guerrero A. R. (2012). Transplantation of mesenchymal stem cells promotes an alternative pathway of macrophage activation and functional recovery after spinal cord injury. *Journal of Neurotrauma*.

[14] Ylostalo J. H., Bartosh T. J., Coble K., Prockop D. J. (2012). Human mesenchymal stem/stromal cells cultured as spheroids are self-activated to produce prostaglandin E2 that directs stimulated macrophages into an anti-inflammatory phenotype. *Stem Cells*.

[15] Anton K., Banerjee D., Glod J. (2012). Macrophage-associated mesenchymal stem cells assume an activated, migratory, pro-inflammatory phenotype with increased IL-6 and CXCL10 secretion. *PLoS One*.

[16] Rahmat Z., Jose S., Ramasamy R., Vidyadaran S. (2013). Reciprocal interactions of mouse bone marrow-derived mesenchymal stem cells and BV2 microglia after lipopolysaccharide stimulation. *Stem Cell Research & Therapy*.

[17] Jacobs S. A., Roobrouck V. D., Verfaillie C. M., Van Gool S. W. (2013). Immunological characteristics of human mesenchymal stem cells and multipotent adult progenitor cells. *Immunology and Cell Biology*.

[18] Jiang Y., Jahagirdar B. N., Reinhardt R. L. (2002). Pluripotency of mesenchymal stem cells derived from adult marrow. *Nature*.

[19] Mora-Lee S., Sirerol-Piquer M. S., Gutierrez-Perez M. (2012). Therapeutic effects of hMAPC and hMSC transplantation after stroke in mice. *PLoS One*.

[20] Sindberg G. M., Lindborg B. A., Wang Q. (2014). Comparisons of phenotype and immunomodulatory capacity among rhesus bone-marrow-derived mesenchymal stem/stromal cells, multipotent adult progenitor cells, and dermal fibroblasts. *Journal of Medical Primatology*.

[21] Bedi S. S., Hetz R., Thomas C. (2013). Intravenous multipotent adult progenitor cell therapy attenuates activated microglial/macrophage response and improves spatial learning after traumatic brain injury. *Stem Cells Translational Medicine*.

[22] DePaul M. A., Palmer M., Lang B. T. (2015). Intravenous multipotent adult progenitor cell treatment decreases inflammation leading to functional recovery following spinal cord injury. *Scientific Reports*.

[23] Walker P. A., Bedi S. S., Shah S. K. (2012). Intravenous multipotent adult progenitor cell therapy after traumatic brain injury: modulation of the resident microglia population. *Journal of Neuroinflammation*.

[24] Busch S. A., Hamilton J. A., Horn K. P. (2011). Multipotent adult progenitor cells prevent macrophage-mediated axonal dieback and promote regrowth after spinal cord injury. *The Journal of Neuroscience*.

[25] Ravanidis S., Bogie J. F., Donders R. (2015). Neuroinflammatory signals enhance the immunomodulatory and neuroprotective properties of multipotent adult progenitor cells. *Stem Cell Research & Therapy*.

[26] Jellema R. K., Ophelders D. R., Zwanenburg A. (2015). Multipotent adult progenitor cells for hypoxic-ischemic injury in the preterm brain. *Journal of Neuroinflammation*.

[27] Subramanian K., Geraerts M., Pauwelyn K. A. (2010). Isolation procedure and characterization of multipotent adult progenitor cells from rat bone marrow. *Methods in Molecular Biology*.

[28] Hendriks J. J., Slaets H., Carmans S. (2008). Leukemia inhibitory factor modulates production of inflammatory mediators and myelin phagocytosis by macrophages. *Journal of Neuroimmunology*.

[29] Lane K. B., Egan B., Vick S., Abdolrasulnia R., Shepherd V. L. (1998). Characterization of a rat alveolar macrophage cell line that expresses a functional mannose receptor. *Journal of Leukocyte Biology*.

[30] Bogie J. F., Stinissen P., Hellings N., Hendriks J. J. (2011). Myelin-phagocytosing macrophages modulate autoreactive T cell proliferation. *Journal of Neuroinflammation*.

[31] Croitoru-Lamoury J., Lamoury F. M., Zaunders J. J., Veas L. A., Brew B. J. (2007). Human mesenchymal stem cells constitutively express chemokines and chemokine receptors that can be upregulated by cytokines, IFN-beta, and Copaxone. *Journal of Interferon & Cytokine Research*.

[32] Schneider C. A., Rasband W. S., Eliceiri K. W. (2012). NIH image to ImageJ: 25 years of image analysis. *Nature Methods*.

[33] Untergasser A., Cutcutache I., Koressaar T. (2012). Primer3—new capabilities and interfaces. *Nucleic Acids Research*.

[34] Livak K. J., Schmittgen T. D. (2001). Analysis of relative gene expression data using real-time quantitative PCR and the 2(−delta delta C(T)) method. *Methods*.

[35] Vandesompele J., De Preter K., Pattyn F. (2002). Accurate normalization of real-time quantitative RT-PCR data by geometric averaging of multiple internal control genes. *Genome Biology*.

[36] Linares D., Taconis M., Mana P. (2006). Neuronal nitric oxide synthase plays a key role in CNS demyelination. *The Journal of Neuroscience*.

[37] Nemeth K., Leelahavanichkul A., Yuen P. S. (2009). Bone marrow stromal cells attenuate sepsis via prostaglandin E(2)-dependent reprogramming of host macrophages to increase their interleukin-10 production. *Nature Medicine*.

[38] Ren G. W., Zhang L. Y., Zhao X. (2008). Mesenchymal stem cell-mediated immunosuppression occurs via concerted action of chemokines and nitric oxide. *Cell Stem Cell*.

[39] Jones T. B., Basso D. M., Sodhi A. (2002). Pathological CNS autoimmune disease triggered by traumatic spinal cord injury: implications for autoimmune vaccine therapy. *The Journal of Neuroscience*.

[40] Kil K., Zang Y. C., Yang D. (1999). T cell responses to myelin basic protein in patients with spinal cord injury and multiple sclerosis. *Journal of Neuroimmunology*.

[41] Tugal D., Liao X., Jain M. K. (2013). Transcriptional control of macrophage polarization. *Arteriosclerosis, Thrombosis, and Vascular Biology*.

[42] Becher B., Bechmann I., Greter M. (2006). Antigen presentation in autoimmunity and CNS inflammation: how T lymphocytes recognize the brain. *Journal of Molecular Medicine (Berlin)*.

[43] Jin R., Yang G., Li G. (2010). Inflammatory mechanisms in ischemic stroke: role of inflammatory cells. *Journal of Leukocyte Biology*.

[44] Katsumoto A., Lu H., Miranda A. S., Ransohoff R. M. (2014). Ontogeny and functions of central nervous system macrophages. *Journal of Immunology*.

[45] Selmaj K. W., Raine C. S. (1988). Tumor necrosis factor mediates myelin and oligodendrocyte damage in vitro. *Annals of Neurology*.

[46] Beck J., Rondot P., Catinot L., Falcoff E., Kirchner H., Wietzerbin J. (1988). Increased production of interferon gamma and tumor necrosis factor precedes clinical manifestation in multiple sclerosis: do cytokines trigger off exacerbations?. *Acta Neurologica Scandinavica*.

[47] Li Q., Sun W., Wang X., Zhang K., Xi W., Gao P. (2015). Skin-derived mesenchymal stem cells alleviate atherosclerosis via modulating macrophage function. *Stem Cells Translational Medicine*.

[48] Spaggiari G. M., Abdelrazik H., Becchetti F., Moretta L. (2009). MSCs inhibit monocyte-derived DC maturation and function by selectively interfering with the generation of immature DCs: central role of MSC-derived prostaglandin E2. *Blood*.

[49] Dayan V., Yannarelli G., Billia F. (2011). Mesenchymal stromal cells mediate a switch to alternatively activated monocytes/macrophages after acute myocardial infarction. *Basic Research in Cardiology*.

[50] Zhang Q. Z., Su W. R., Shi S. H. (2010). Human gingiva-derived mesenchymal stem cells elicit polarization of m2 macrophages and enhance cutaneous wound healing. *Stem Cells*.

[51] Ren G., Zhao X., Zhang L. (2010). Inflammatory cytokine-induced intercellular adhesion molecule-1 and vascular cell adhesion molecule-1 in mesenchymal stem cells are critical for immunosuppression. *Journal of Immunology*.

[52] Duffy M. M., Pindjakova J., Hanley S. A. (2011). Mesenchymal stem cell inhibition of T-helper 17 cell-differentiation is triggered by cell-cell contact and mediated by prostaglandin E2 via the EP4 receptor. *European Journal of Immunology*.

[53] Xing Z., Gauldie J., Cox G. (1998). IL-6 is an antiinflammatory cytokine required for controlling local or systemic acute inflammatory responses. *The Journal of Clinical Investigation*.

[54] Gallucci R. M., Simeonova P. P., Matheson J. M. (2000). Impaired cutaneous wound healing in interleukin-6-deficient and immunosuppressed mice. *The FASEB Journal*.

[55] Roca H., Varsos Z. S., Sud S., Craig M. J., Ying C., Pienta K. J. (2009). CCL2 and interleukin-6 promote survival of human CD11b+ peripheral blood mononuclear cells and induce M2-type macrophage polarization. *The Journal of Biological Chemistry*.

[56] Bouffi C., Bony C., Courties G., Jorgensen C., Noël D. (2010). IL-6-dependent PGE2 secretion by mesenchymal stem cells inhibits local inflammation in experimental arthritis. *PLoS One*.

[57] di Penta A., Moreno B., Reix S. (2013). Oxidative stress and proinflammatory cytokines contribute to demyelination and axonal damage in a cerebellar culture model of neuroinflammation. *PLoS One*.

[58] Krampera M. (2011). Mesenchymal stromal cell ‘licensing’: a multistep process. *Leukemia*.

[59] Erwig L. P., Henson P. M. (2008). Clearance of apoptotic cells by phagocytes. *Cell Death and Differentiation*.

[60] Reading J. L., Vaes B., Hull C. (2015). Suppression of IL-7-dependent effector T-cell expansion by multipotent adult progenitor cells and PGE2. *Molecular Therapy*.

[61] Donders R., Vanheusden M., Bogie J. F. (2014). Human Wharton’s jelly-derived stem cells display immunomodulatory properties and transiently improve rat experimental autoimmune encephalomyelitis. *Cell Transplantation*.

[62] Neirinckx V., Agirman G., Coste C. (2015). Adult bone marrow mesenchymal and neural crest stem cells are chemoattractive and accelerate motor recovery in a mouse model of spinal cord injury. *Stem Cell Research & Therapy*.

[63] Eggenhofer E., Luk F., Dahlke M. H., Hoogduijn M. J. (2014). The life and fate of mesenchymal stem cells. *Frontiers in Immunology*.

[64] David S., Greenhalgh A. D., Kroner A. (2015). Macrophage and microglial plasticity in the injured spinal cord. *Neuroscience*.

[65] Hsieh C. L., Kim C. C., Ryba B. E. (2013). Traumatic brain injury induces macrophage subsets in the brain. *European Journal of Immunology*.

[66] Bogie J. F., Jorissen W., Mailleux J. (2013). Myelin alters the inflammatory phenotype of macrophages by activating PPARs. *Acta Neuropathologica Communications*.

[67] Miron V. E., Boyd A., Zhao J. W. (2013). M2 microglia and macrophages drive oligodendrocyte differentiation during CNS remyelination. *Nature Neuroscience*.

[68] van Zwam M., Samsom J. N., Nieuwenhuis E. E. (2011). Myelin ingestion alters macrophage antigen-presenting function in vitro and in vivo. *Journal of Leukocyte Biology*.

[69] Wagner B., Henschler R. (2013). Fate of intravenously injected mesenchymal stem cells and significance for clinical application. *Advances in Biochemical Engineering/Biotechnology*.

[70] Hess D. C., Sila C. A., Furlan A. J., Wechsler L. R., Switzer J. A., Mays R. W. (2014). A double-blind placebo-controlled clinical evaluation of MultiStem for the treatment of ischemic stroke. *International Journal of Stroke*.

