# Supplementary material for: Crosstalk with Inflammatory Macrophages Shapes the Regulatory Properties of Multipotent Adult Progenitor Cells
Source: Stem Cells Int. 2017 Jul 12;2017:2353240. doi: 10.1155/2017/2353240 (PMC5529661; doi:10.1155/2017/2353240)
Supplement: Supplementary file 1 — Supplemental figures Supplemental Figure 1: Schematic illustration of generation of conditioned media from rMAPC and application to macrophages (MΦ). Supplemental Figure 2: Macrophage-primed rMAPC increase mRNA expression of chemokines. CCL2, CCL5 and CXCL10 mRNA expression in rMAPC treated with SN of LPS-activated macrophages, SN of naïve macrophages (-LPS-SN) or LPS. Results are shown as fold differences in comparison to SN of naïve macrophages. Expression of target genes was normalized against expression of 14-3-3 protein zeta/delta (YWHAZ) and TATA-binding protein (TBP). Mean values ± SEM are from 5 experiments. Asterisks (∗) indicate statistical significant difference with SN of naïve macrophages. Data were analyzed with one way ANOVA followed by Dunnett's multiple comparison test. ∗p≤0.05, ∗∗p≤0.01 and ∗∗∗p≤0.001. [file 2353240.f1.docx]

**Supplemental table 1:** Sequences of primers used for qPCR

| gene | forward primer (5’-3’) | reverse primer (5’-3’) | product (in bp) |
| --- | --- | --- | --- |
| *iNOS* | GCATCCCAAGTACGAGTGGT | TGTTGTAGCGCTGTGTGTCA | 176 |
| *COX-2* | TCACATTTGATTGACAGCCC | CCTTATTTCCTTTCACACCCA | 148 |
| *IFN-γ* | GAAAGACAACCAGGCCATCAG | TCATGAATGCATCCTTTTTTGC | 101 |
| *TNF-α* | CTTATCTACTCCCAGGTTCTCTTCAA | GAGACTCCTCCCAGGTACATGG | 200 |
| *IL-6* | TAGTCCTTCCTACCCCAACTTCC | TTGGTCCTTAGCCACTCCTTC | 76 |
| *Arg1* | CAAGCTGGGAATTGGCAAAG | GGTCCAGTCCATCAACATCAAA | 101 |
| *CD86* | TCCATCAGCCTATCTCTTCCATTC | GCTGCGAAAAAACCATATTGTG | 118 |
| *CCR1* | TCTGGAAACACAGACTCATTGTC | CCACTCCAATGATAAACACGAA | 227 |
| *CCR3* | AAACCTGAGAAGCTAGCCTGTTT | TGCCATTTTACTTGTCTCTGATG | 163 |
| *CXCL10* | GGGCCATAGGAAAACTTGAAATC | CATTGTGGCAATGATCTCAACAT | 71 |
| *CCL2* | TGTCTCAGCCAGATGCAGTT | TTCCTTATTGGGGTCAGCAC | 179 |
| *CCL5* | CCTTGCAGTCGTCTTTGTCA | ATCCCCAGCTGGTTAGGACT | 175 |
| *CCL18* | CAAGAAGAGATGGCTGCCAGTC | CGATTGTGATGGTTCGAAGTGA | 95 |
| Reference genes |  |  |  |
| *RPL13* | GGATCCCTCCACCCTATGACA | CTGGTACTTCCACCCGACCTC | 131 |
| *TBP* | TGGGATTGTACCACAGCTCCA | CTCATGATGACTGCAGCAAACC | 132 |
| *CycA* | TATCTGCACTGCCAAGACTGAGTG | CTTCTTGCTGGTCTTGCCATTCC | 127 |
| *YWHAZ* | GATGAAGCCATTGCTGAACTTG | GTCTCCTTGGGTATCCGATGTC | 117 |
| *PGK-1* | ATGCAAAGACTGGCCAAGCTAC | AGCCACAGCCTCAGCATATTTC | 104 |
| *HPRT* | CTCATGGACTGATTATGGACAGGAC | GCAGGTCAGCAAAGAACTTATAGCC | 123 |
| *b-actin* | TGTCACCAACTGGGACGATA | GGGGTGTTGAAGGTCTCAAA | 165 |
| *HMBS* | TCCTGGCTTTACCATTGGAG | TGAATTCCAGGTGAGGGAAC | 176 |
